# The Final Frontier of Sustainable Materials: Current Developments in Self-Healing Elastomers

**DOI:** 10.3390/ijms23094757

**Published:** 2022-04-26

**Authors:** Saul Utrera-Barrios, Raquel Verdejo, Miguel Ángel López-Manchado, Marianella Hernández Santana

**Affiliations:** Institute of Polymer Science and Technology (ICTP-CSIC), Juan de la Cierva 3, 28006 Madrid, Spain; sutrera@ictp.csic.es (S.U.-B.); r.verdejo@csic.es (R.V.)

**Keywords:** self-healing materials, self-healing rubbers, natural rubber, synthetic rubber, dynamic networks, supramolecular chemistry

## Abstract

It is impossible to describe the recent progress of our society without considering the role of polymers; however, for a broad audience, “*polymer*” is usually related to environmental pollution. The poor disposal and management of polymeric waste has led to an important environmental crisis, and, within polymers, plastics have attracted bad press despite being easily reprocessable. Nonetheless, there is a group of polymeric materials that is particularly more complex to reprocess, rubbers. These macromolecules are formed by irreversible crosslinked networks that give them their characteristic elastic behavior, but at the same time avoid their reprocessing. Conferring them a self-healing capacity stands out as a decisive approach for overcoming this limitation. By this mean, rubbers would be able to repair or restore their damage automatically, autonomously, or by applying an external stimulus, increasing their lifetime, and making them compatible with the circular economy model. Spain is a reference country in the implementation of this strategy in rubbery materials, achieving successful self-healable elastomers with high healing efficiency and outstanding mechanical performance. This article presents an exhaustive summary of the developments reported in the previous 10 years, which demonstrates that this property is the last frontier in search of truly sustainable materials.

## 1. Introduction

In the actual environmental context, polymers like rubbers are particularly critical due to their reprocessing difficulties. These macromolecular materials are composed of irreversible crosslinked networks that act as “*anchor points*”, preventing the flow of polymeric chains. Consequently, the material cannot be reshaped [1], and a considerable amount of rubber waste could be generated. One of the strategies to solve this issue has been the recovery of end-of-life rubbers for their use as a diluent or reinforcing filler in new composite materials [2,3,4,5,6]. Also, the selective breaking of the crosslinking points, known as devulcanization [7,8,9,10,11], has been extensively studied; however, both strategies are considered insufficient. Thus, the redesign of crosslinked rubbers is mandatory. Most recent redesign strategies point toward building dynamic networks [1,12,13].

The creation of crosslinked polymers with dynamic networks has spawned a new generation of polymers known as DYNAMERS (*DYNAmic polyMERS*) [14,15]. The construction of these networks is based on multiple dynamic bonds and/or supramolecular interactions, like hydrogen bonds [16,17], ionic interactions [18], metal–ligand coordination [19], disulfide exchange [20], and Diels–Alder chemistry [21,22], among other covalent, non-covalent mechanisms and/or combinations between them [23,24,25,26,27,28,29,30]. The reversible nature of these networks can be controlled by an external stimulus, which can be temperature, pressure, electrical current, magnetic field, or further changes in the medium, such as pH [31,32,33,34,35]. In this way, the stimuli-responsive material would be able to release its “*anchor points*”, allowing the flow of its chains until it reforms and/or repairs. In fact, the use of dynamic networks is the most widespread self-healing strategy used in rubbers [1].

**Figure 1:**
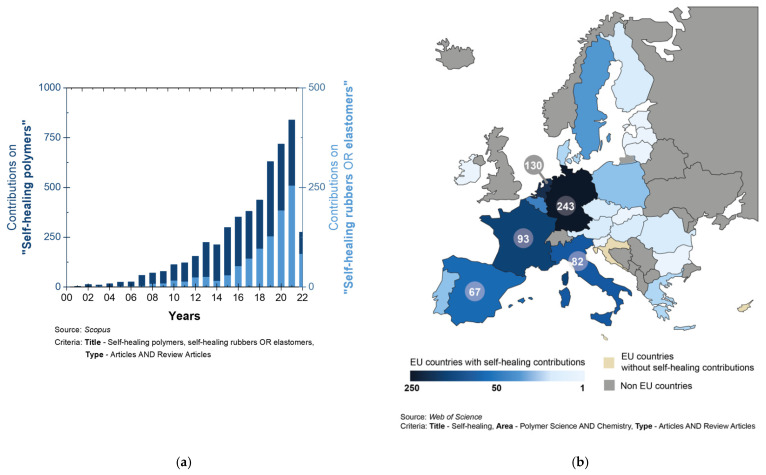
(**a**) Self-healing polymers and elastomers contributions in the 21st century, (**b**) Self-healing polymers contributions in European Union countries (complete United Kingdom data excluded). Source: *Web of Science*.

The year 2001 is considered the starting date for the development of self-healing materials as we know them today [36]. A self-healable material can repair or restore its damage automatically, autonomously, or through the application of an external stimulus. Such a concept has been incorporated in concrete and cement [37], asphalt [38], metals [39], polymers [40], and composite materials [41,42,43]. Despite the multiple strategies involved in each family of materials, they all have a common goal: a more sustainable future, by increasing their lifetime and reducing waste. Research on self-healing polymers and specifically on self-healing rubbers or elastomers has not stopped growing since then (Figure 1a) [40,44]. Spain has played a leading role in this growth, positioning itself within the Top 5 of the European Union countries that contribute the most (Figure 1b). This article presents an exhaustive review of said scientific contributions over the last 10 years (2012–2022), classified according to the nature of the matrices involved (natural or synthetic), as well as the main challenges and perspectives of these developments.

## 2. To Boldly Go Where No Material Has Gone before: Self-Healing Concepts

**Figure 2:**
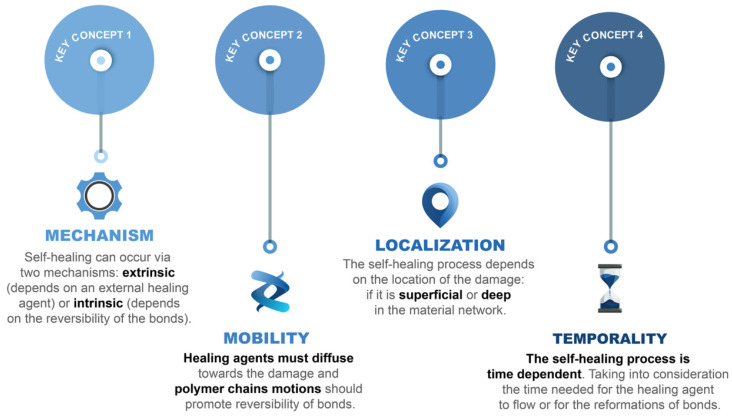
Self-healing key concepts. Adapted with permission from [49]. 2020, Royal Society of Chemistry.

Self-healing is the ability to repair or restore damages [45,46,47,48]. To scientifically understand healing as a physical process, four key concepts must be considered in rubbers (Figure 2): (1) Mechanism, (2) Mobility, (3) Localization and (4) Temporality [49]. In elastomers, the success of the self-healing process goes hand in hand with the adequate selection of a mechanism that guarantees the necessary molecular mobility of the polymeric chains, as well as enough time for the restoration of the damage according to its location (on a macroscopic or microscopic scale) [50].

The first concept is the *mechanism*. Self-healing can occur extrinsically or intrinsically [51,52]. Extrinsic mechanisms are based on an external healing agent that is incorporated into the matrix in an encapsulated form, in vascular networks or freely dispersed. When damage occurs, these agents are released and/or flow through the damage area, sealing it. Despite being the first mechanisms used, according to the historical development, their use in rubbers is very limited due to the difficulties of preserving the stability of the agent during the conventional mixing process of rubber recipes (enormous shear forces) [49].

**Figure 3:**
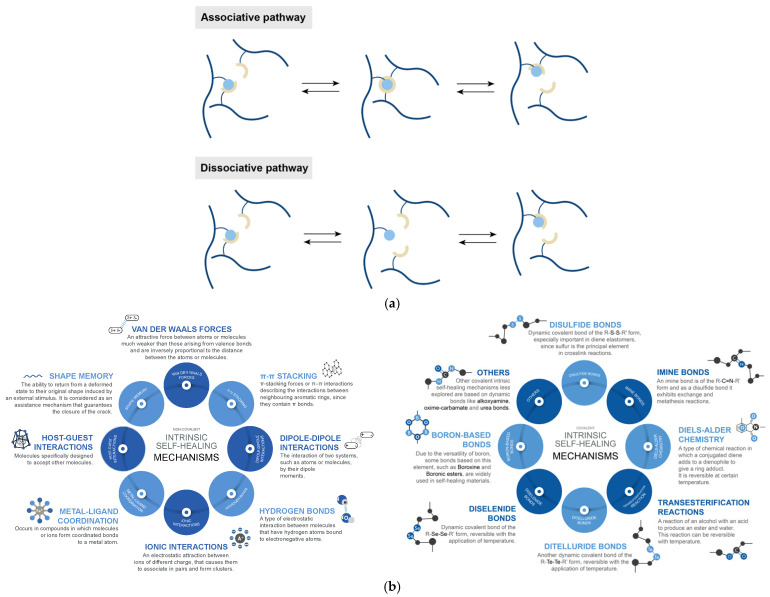
(**a**) Pathways of covalent exchange reactions, (**b**) Intrinsic self-healing mechanisms. Adapted with permission from [49,53]. 2021, Elsevier (**a**) and 2020, Royal Society of Chemistry (**b**).

The intrinsic mechanisms are based on the creation of crosslinking points using dynamic covalent bonds or supramolecular interactions. On one hand, dynamic covalent bonds activate this character under different external stimuli and can occur through an associative or dissociative pathway (Figure 3a). The associative pathway is characterized by a constant crosslink density during the exchange. Meanwhile, the dissociative one is characterized by a change in the crosslink density over time, due to an independent reformation and formation of the bonds [15,53]. On the other hand, supramolecular interactions are non-covalent in nature and have also been shown to be successful strategies to achieve repairability [15,54,55].

From a general point of view, intrinsic mechanisms can be classified as [51] (Figure 3b):

1. Non-covalent intrinsic mechanisms, such as hydrogen bonds, ionic interactions, metal–ligand coordination, among others; and,
2. Covalent intrinsic mechanisms, such as disulfide bond exchange (associative), Diels–Alder chemistry (dissociative), transesterification reactions (associative), bonds based on boron and imines chemistry (dissociative), among others.

In recent years, the creation of hybrid networks by multiple combinations of covalent and non-covalent mechanisms has become remarkably widespread [1,49].

The second concept is *mobility*. It is a priority concept for self-healing, regardless of the mechanism. In the case of the intrinsic ones, the mobility of the chains will be mandatory to guarantee the success of the exchange reactions. If the rubber network is very impeded, more severe conditions are required (e.g., high temperatures) that could seriously compromise the stability of the material [49].

The third concept is *localization*. It is related to the scale of the damage. According to the literature, repair on a microscopic scale is easier than on a macroscopic one. This is completely expected from the physical point of view. In addition, this localization will also have a considerable influence on the repair conditions. At larger scales, the required repair conditions will be more severe [49,50].

The last concept is temporality. Ideally, self-healing would be an automatic process, but in practice, it is time-dependent. This dependence is also strongly related to the external stimulus used. One of the greatest complexities of self-healing as a scientific strategy stems from the consideration of all these variables and conditions that must be exhaustively optimized to guarantee a compromise between self-healing capacity, mechanical performance, and material integrity [49].

## 3. Current Developments in Self-Healing Elastomers

**Figure 4:**
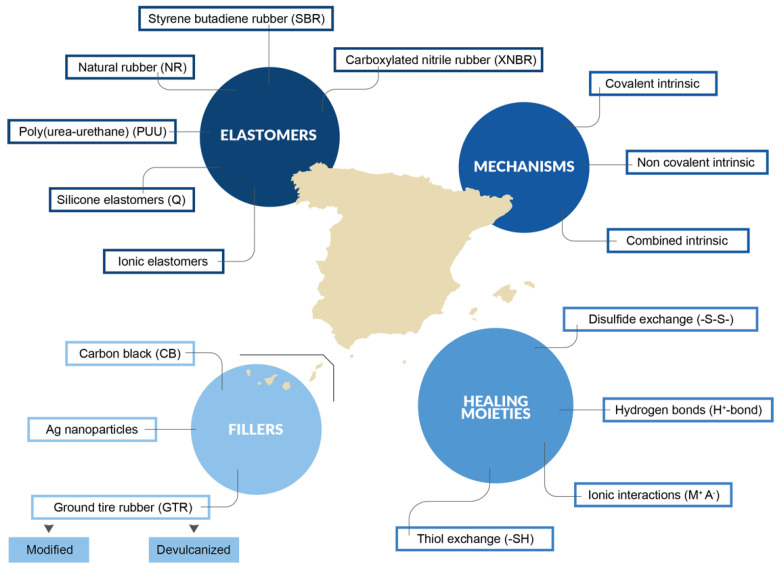
Self-healing elastomers contributions in Spain.

In this section, we summarize the most recent developments on self-healing natural and synthetic elastomers done by research groups in Spain (Figure 4). We thoroughly discuss the underlying healing mechanisms, as well as the challenges and possibilities of applying them to real-life applications.

### 3.1. Self-Healing Natural Rubber

**Figure 5:**
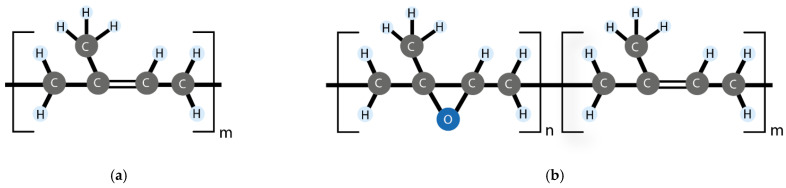
(**a**) NR chemical structure, (**b**) ENR chemical structure.

Natural rubber (NR) consists of cis-1,4-polyisoprene chains [56]. It is the only natural macromolecule completely constituted by carbon (C) and hydrogen (H) atoms (Figure 5a), obtained from multiple varieties of plants and fungi, where the most commercially representative is the Hevea Brasiliensis tree [57]. NR is characterized by having high elasticity, even in the unvulcanized state, due to a naturally occurring network of the non-rubber components, which is responsible for its green strength and facilitates the strain-induced crystallization behavior characteristic of this material [58,59,60,61,62,63].

NR has its origin in South America, but currently, the largest production is concentrated in Southeast Asia, with Thailand and Indonesia concentrating more than 50% of the world’s production. Among its most common applications is the manufacture of large tires, especially for aircraft, vehicles, and heavy machinery, as well as bridge mounts, anti-vibration devices, conveyor belts, and other high-performance elastomeric parts [56].

Due to the demands of its processing, NR is not among the most studied rubbers for self-healing; however, Spanish scientists were pioneers in the study of this material, taking advantage of the existence of sulfur crosslinked points that can serve as healing moieties. Table 1 shows the studies available in the literature.

The first approximation of a self-healing NR was presented by Hernández Santana et al. [67,68]. In this study, the authors took advantage of an ingredient present in most NR formulations: sulfur. Sulfur is the crosslinking agent *par excellence* in most diene elastomers. Its combination with other ingredients, such as accelerants and activators, enables networks *on demand*. By varying the sulfur/accelerant ratio, a complete control can be achieved over the type of crosslinks formed: monosulfide, disulfide and polysulfide. The last two can be used as healing moieties, considering their exchange reactions. In these preliminary studies, healing efficiencies of up to 80% were achieved with a protocol of 70 °C for 7 h.

**Figure 6:**
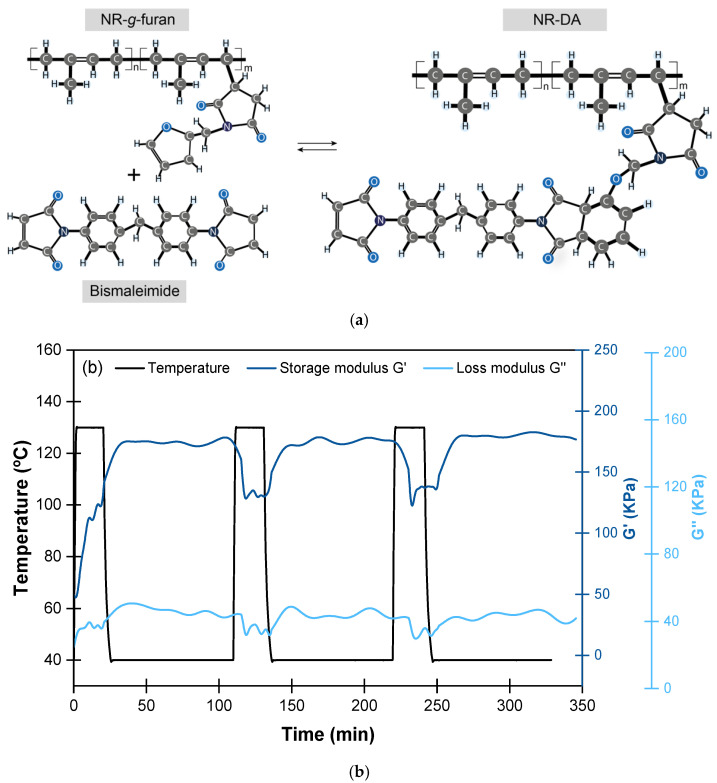
(**a**) DA-based crosslinked process, (**b**) dynamic character of the DA reaction evidenced by DMA. Adapted with permission from [64]. 2019, Elsevier.

The first NR study fully conducted in Spain was that of Tanasi et al. [64]. In this research, a major challenge was posed: the functionalization of NR to enable its crosslinking through the well-known Diels–Alder chemistry (NR-DA). For this, furan groups (dienes) were incorporated into malleated NR structure (NR-*g*-Furan) that were crosslinked with bismaleimide (dienophile) (Figure 6a). This strategy enabled the recovery of up to 80% of the modules at low and medium strains with a combined protocol of 130 °C for 4 h (for the retro-DA reaction) followed by 40 °C for 7 days (for the reformation of the DA adduct). The reversibility of the DA reaction was confirmed through mechano-dynamic analysis (DMA), evidencing the recovery of the storage (G′) and loss (G″) moduli after three cycles (Figure 6b).

Attempts have also been made to take advantage of some modified NR variants that have different reactivity and are usually more aging-resistant (due to the decrease in double bonds). One variant is epoxidized natural rubber (ENR), which results from the reaction between the NR and a peracid [69]. Such reaction modifies the chemical structure inserting epoxy groups that can vary commercially between 25 mol% and 50 mol% (Figure 5b) [70], but they can also be synthesized in the laboratory with different contents [71]. ENR is among the most explored self-healing rubbers [65,72,73,74,75,76].

Utrera-Barrios et al. [65] reported the vulcanization of this material with very low dicumyl peroxide (DCP) contents, enabling self-healing after 24 h at room temperature under pressure. In this study, two different commercial ENR were used, observing that the self-healing capacity increases with the content of epoxy units thanks to the formation of hydrogen bonds between these and their hydroxyl and carboxyl derivatives during vulcanization. At the same time, the inverse character of the healing efficiency with the crosslink density was shown; at contents higher than 0.8 phr of the crosslinking agent (DCP), it was not possible to achieve acceptable self-healing values. Thanks to the ease of mixing NR and its derivatives with different fillers systems, in this same study, the incorporation of graphene oxide (GO) selectively functionalized with hydroxyl and carboxyl groups was tested to enhance the formation of hydrogen bonds with the matrix. With this strategy, it was possible to increase the healing efficiency from 50% (of pure ENR) to 85%, demonstrating the potential of manufacturing elastomeric composite materials (compounds) where the filler also contributes to the self-healing mechanism.

**Figure 7:**
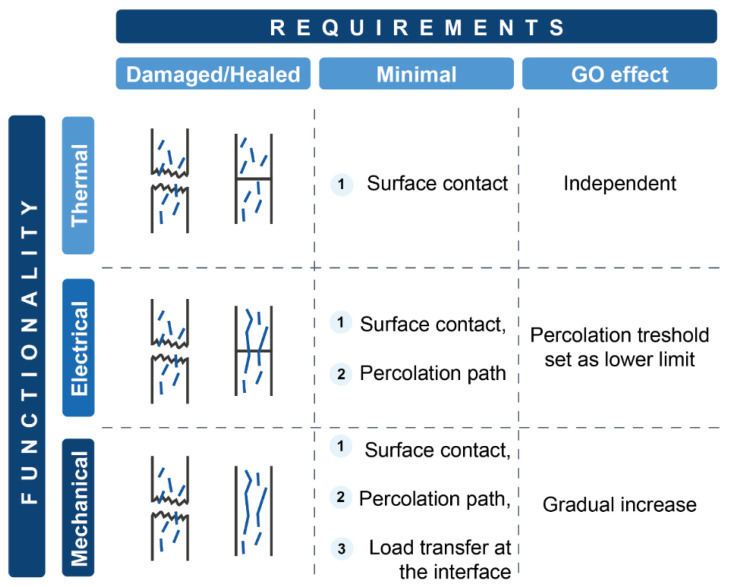
Effect of GO on mechanical, thermal, and electrical healing of NR. Adapted with permission from [66]. 2017, IOP Science.

GO was also incorporated into NR by Hernández Santana et al. [66], to observe its influence on the recovery of mechanical, thermal, and electrical functionalities. The healing efficiency from the mechanical point of view was studied as the retention of the properties at the failure point of a tensile test; the electrical healing efficiency was determined as the recovery of the electrical conductivity of the material through dielectric spectroscopy studies and, finally, the efficiency of thermal healing was quantified as the recovery of the thermal conductivity. It was observed that both the mechanical and electrical healing depend on the GO content. Figure 7 summarizes the most important considerations in each case and the results obtained.

All these strategies have proven to be very effective from the point of view of healing efficiency; however, much remains to be done to increase the mechanical performance of these materials. This is mandatory for allowing the industrial scalability of NR to real applications that usually demand superior performance.

### 3.2. Self-Healing Synthetic Elastomers

Most commercially relevant elastomers are of synthetic origin, representing over 55% of world production [77]. From a basic point of view, synthetic rubbers and elastomers have been created to replace NR in those scenarios where it does not perform well: at high and low temperatures, outdoors, in contact with petroleum-derived solvents, as well as to avoid gas permeability. Styrene-butadiene rubber (SBR), carboxylated nitrile rubber (XNBR), silicone elastomers and poly(urea-urethanes) (PUU), are some examples. Table 2 shows the studies available in the literature on self-healing materials (identified as elastomers or rubbers by their authors).

**Figure 8:**
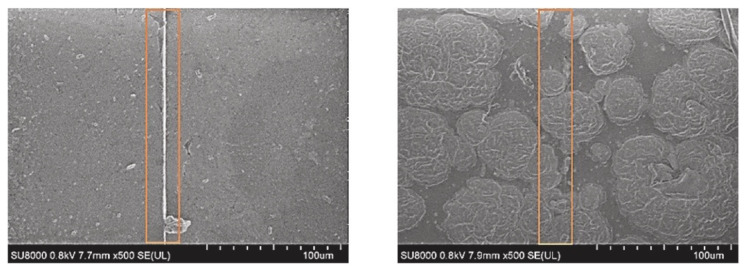
SEM micrograph of sulfur-based SBR compound before (**left**) and after (**right**) healing protocol. Reproduced with permission from [50]. 2022, Elsevier.

**Figure 9:**
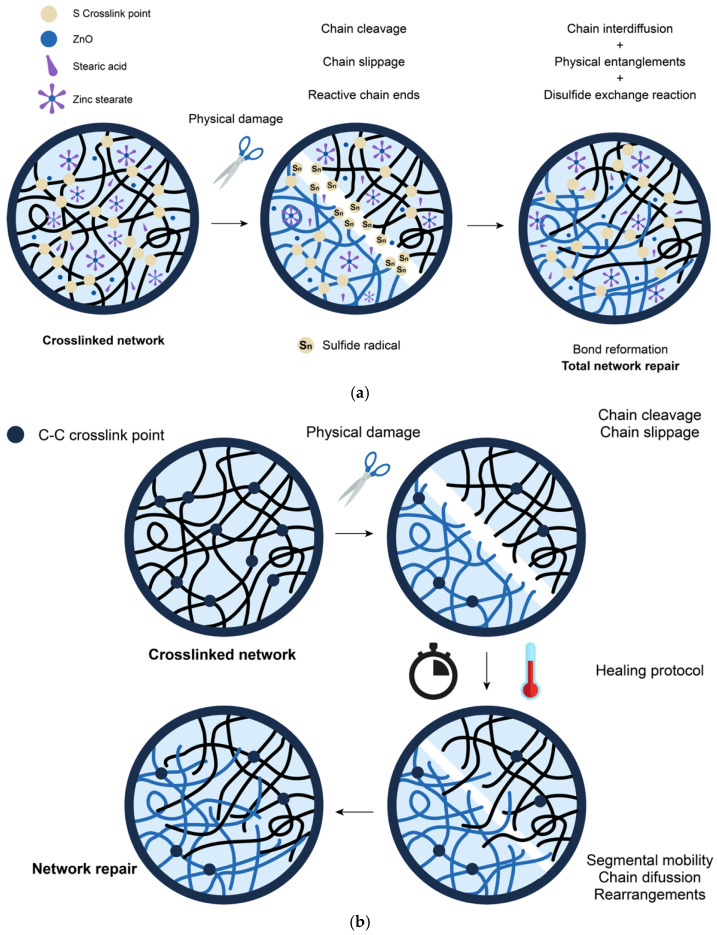
Schematic representation of self-healing process in (**a**) sulfur-based and (**b**) peroxide-based SBR. Adapted with permission from [50]. 2022, Elsevier.

Among all the synthetic variants, SBR is the one with the highest production and consumption due to its extended use in tire treads. Araujo-Morera et al. [50] presented an exhaustive study on the influence of the different ingredients of a rubber formulation on the self-healing capacity of SBR. They studied the influence of the accelerant/sulfur ratio, the nature of the vulcanizing agent (sulfur vs. peroxide), as well as the content of activators (zinc oxide and stearic acid). All ingredients were shown to play a determining role in the self-healing capacity of SBR. The evolution of a crack was followed by scanning electron microscopy (SEM), observing its complete disappearance (fully visual recovery) after 1 h at 130 °C (Figure 8). With an optimal content of 1 phr of sulfur, the best healing efficiency of up to 80% was achieved thanks to disulfide exchange reactions (Figure 9a). In addition, self-healing in a peroxide crosslinked SBR compound was reported for the first time, which was attributed to the early stages of the repair process, where the interdiffusion of the polymer chains allows a partial recovery of the material’s properties (Figure 9b). This initial study served as the basis for the incorporation of ground tire rubber (GTR) derived from ELTs as a sustainable strategy, thanks to the possible compatibility of both materials; GTR is normally composed of NR, SBR, butadiene rubber (BR) and butyl rubber (IIR) [84].

Hernández Santana et al. [78] reported for the first time the development of SBR compounds with GTR. The self-healing efficiency of GTR-filled SBR compounds was compared with conventional recipes with carbon black (CB), the most common reinforcing filler in the rubber industry. The results showed that the incorporation of GTR, unlike CB, does not substantially reduce the self-healing capacity of SBR, while improving the mechanical properties by 50%. Thus, the use of a waste material for the development of new elastomeric composites was evidenced as an economically and environmentally sustainable strategy. With the intention of optimizing the role of GTR in SBR compounds, Araujo-Morera et al. [84] continued in this line of research and modified the GTR powder through different mechano-chemical strategies. They reported that, thanks to the modification of the GTR with sulfuric acid (H2SO4) (mGTR), it was possible to incorporate functionalities to the material that improved its compatibility with the rubber, while increasing the reinforcing character and its use as self-healing precursor. The incorporation of just 10 phr of mGTR increased the tensile strength of the starting material by 115%, with enormous potential in self-healing applications.

**Figure 10:**
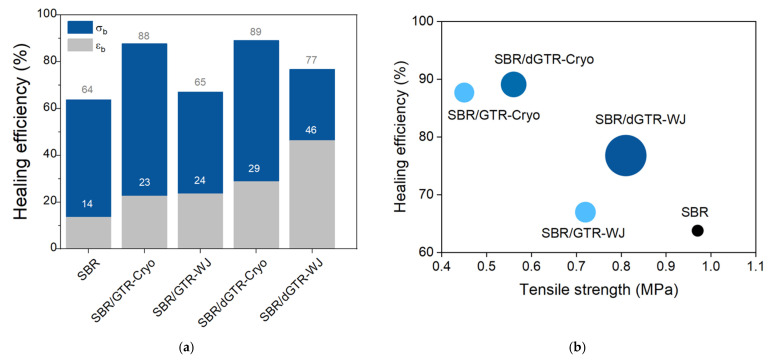
Influence of grinding protocols (cryogenic fracture, Cryo, and water jet, WJ) in the (**a**) healing efficiency and (**b**) tensile strength of SBR/30 phr dGTR compounds. The symbol size is scaled according to the recovery of the elongation at break. Adapted with permission from [85]. 2022.

In this context, Alonso Pastor et al. [85] presented a study related to the use of devulcanized GTR (dGTR). For the first time, this treated material was used as a filler in a self-healing SBR matrix. Different GTR devulcanization mechanisms (thermo-mechanical, microwave and thermo-chemical) were evaluated, as well as different GTR morphologies obtained from cryogenic or water jet grinding processes. It was shown that the combination of a cryogenic grinding with a thermo-mechanical devulcanization protocol enabled the incorporation of up to 30 phr of dGTR, with healing efficiencies of up to 89% (Figure 10a) of the tensile strength (around 0.5 MPa) (Figure 10b). The combination of this grinding process and devulcanization technique provides the highest percentage of free surface polymeric chains, enabling the reformation of new disulfide bridges derived from the residual sulfur present in the dGTR. These conditions were crucial for improving the self-healing behavior.

The recovery of the maximum deformation is also of interest for elastomer-based applications. Thus, the authors considered the evaluation of an overall mechanical healing efficiency, based on the recovery of the tensile strength as well as the recovery of the elongation at break. Figure 10b shows that a better balance was achieved with the water jet devulcanization technique, achieving a tensile strength closer to that of unfilled SBR and still having an overall healing efficiency around 80%.

**Figure 11:**
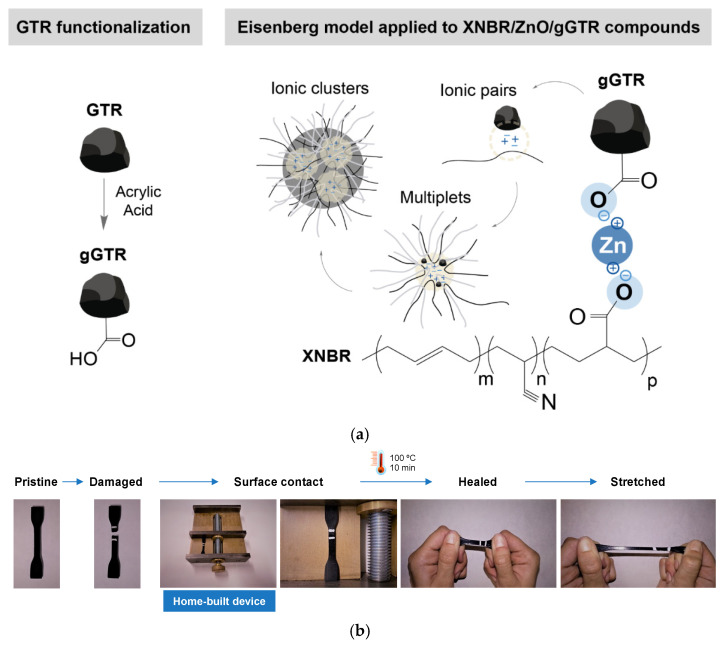
(**a**) Scheme of ionic interactions in XNBR/ZnO/gGTR compounds, (**b**) home-built device used for self-healing tests. Adapted with permission from [79]. 2020, Elsevier.

GTR was also incorporated into a matrix, a priori, incompatible as the XNBR. Utrera-Barrios et al. [79] presented a strategy to improve the compatibility of GTR with XNBR. For this, they functionalized the cryoground GTR powder with carboxylic groups (gGTR), through a poly(acrylic acid) grafting reaction. Carboxylic groups on XNBR and gGTR can form ionic interactions with metal oxides, such as ZnO. In this research, 6 phr of ZnO were incorporated and the formation of ionic pairs that are capable of grouping in supramolecular structures known as multiplets and clusters was demonstrated (Figure 11a). However, the saturation of these groups in the pure rubber prevents self-healing without pressure. By breaking this saturation by incorporating new free carboxylic groups (of gGTR), the self-healing capacity of the XNBR was increased from 10% to 70% in just 10 min at 100 °C. To ensure good contact between the two surfaces during the self-healing process, a home-built device was used. In addition to the good results in terms of mechanical performance, the repaired material exhibited high deformability (Figure 11b) and excellent chemical resistance. This set of good and well-balanced properties makes this XNBR-gGTR compound an excellent candidate for automotive applications with extended lifetime.

Under the same principle as XNBR, other carboxylated elastomers can be crosslinked with metal oxides. However, the carboxylation of elastomers is not only a synthesis approach that enables alternative crosslinking to sulfur; the ionic interactions can also serve as healing moieties with the appropriate choice of a cation [87]. These interactions have enabled the design of numerous synthetic elastomers. Mecerreyes et al. [83] prepared ionic elastomers entirely based on renewable additives. For this, they selected a dimer diamine and various dicarboxylic acids (malonic, citric, tartaric, and 2,5-furandicarboxylic acids) that reacted by means of a proton transfer reaction. Although the self-healing capacity was not quantified, it was qualitatively evaluated, observing a complete recovery of the damaged area, which enabled the material to be stretched up to twice its original length, all this at room temperature and after only 30 min. More recently, novel synthetic elastomers have also been reported. O’Harra et al. [82] presented the development of a polyamide ionene (PAI) elastomer which can be processed by 3D printing and whose healing efficiency is determined by a triple combination of ionic interactions, hydrogen bonds and π-π stacking.

Among all the synthetic elastomers available, polyurethanes (PU) are characterized by their versatility. The numerous combinations between the different types of existing diisocyanates, polyols, diols, and diamines result in materials with a wide range of behaviors spanning from very rigid solids to soft, flexible foams [88]. Elastomeric PU, and particularly PUU, have been addressed in the Spanish self-healing line of research.

Rekondo et al. [81] presented the development of self-healing PUU elastomers thanks to aromatic disulfide exchange reactions. Aromatic disulfide metathesis is one of the few useful covalent reactions for self-healing that can occur at room temperature. The adequate combination of a diisocyanate, a polyol and an aromatic disulfide diamine as a crosslinker enabled the construction of a network with mechanical strengths of up to 0.8 MPa. The combination of the disulfide exchange with a non-covalent hydrogen bonding interaction enabled healing with 80% efficiency in just 2 h, and 100% efficiency after 24 h. These materials also exhibited excellent recyclability [80].

There have also been other important studies related to self-healing in Spain, but not necessarily on rubbers or elastomers, focused on the computational analysis of the mechanisms involved. The use of molecular dynamics techniques has served to complement the understanding of underlying exchange reactions in the presence of a different external stimuli [89,90].

## 4. Challenges, Perspectives and Outlook

**Figure 12:**
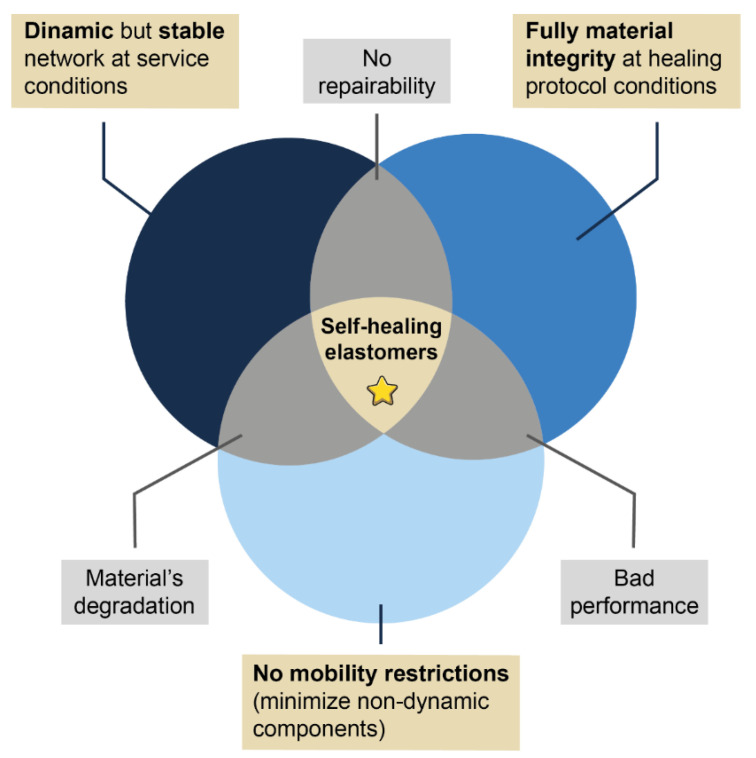
Venn diagram illustrating optimal healing conditions.

In this article, we have shown successful research examples of self-healing elastomers. Until now, different matrices (natural and synthetic) with potential industrial applications have been studied. However, there is still much work to be done. Although it is true that efforts point towards the scalability of self-healing concepts in commercial applications, a comprehensive understanding of the underlying self-healing mechanisms, as well as the optimization of its conditions, is still pending. In the authors’ experience, the redesign of elastomeric compounds to exhibit this capability requires the consideration of three main conditions (Figure 12):

1. The construction of a dynamic but stable and robust network at service temperatures to guarantee excellent mechanical performance;
2. The minimization of components that can hinder the mobility necessary to achieve healing (e.g., secondary irreversible networks); and,
3. The optimization of the appropriate conditions (temporality and external stimulus) for each repair mechanism. In turn, these conditions must be compatible with the material stability, to avoid its deterioration during the healing protocols.

Achieving an optimal compromise between these conditions is vital but, of course, not easy. Furthermore, self-healing is not only about what we do, but also about how we do it. The development of a standardized strategy to quantify repair efficiencies continues to be necessary. Although the measurement of this capacity by retaining the properties at the breaking point of a tensile test seems to be the most widespread path, this is not enough, because there is still a lack of standards that guarantee other aspects beyond a number. Repair protocols must be adapted to the type and location of the damage. The service conditions also should be considered. This is the only way to achieve a fair comparison between different healable materials.

On this road, it is important to not overlook the economic viability, the commercial prospect, and the corresponding life cycle assessment (LCA) that corroborates its environmental impact. When all these conditions are matched, the massive scalability of self-healing materials will be an irreversible fact, and we will have taken one of the definitive steps towards the consolidation of a truly sustainable society. Without a doubt, small steps are currently being taken in this direction, and Spain is ready to continue collaborating with corporations, government entities, and other bodies responsible for the rubber production chain, through the leadership of this long and winding, but exciting, road.

## Figures and Tables

**Table 1:** Self-healing natural rubber research conducted in Spain (2012–2022).

Matrix	Mechanism	Healing Moieties	Filler	Reference
NR	Covalent intrinsic	Diels–Alder chemistry	Unfilled	[64]
ENR	Non-covalent intrinsic	Hydrogen bonds	Unfilled	[65]
NR	Covalent intrinsic	Disulfide exchange	Graphene oxide	[66]
ENR	Combined intrinsic	Hydrogen bonds + Transesterification reactions	Graphene oxide	[65]

**Table 2:** Self-healing synthetic elastomers research conducted in Spain (2012–2022).

Matrix	Mechanism	Healing Moieties	Filler	Reference
SBR	Covalent intrinsic	Disulfide exchange	Unfilled	[50,78]
XNBR	Non-covalent intrinsic	Ionic interactions	Unfilled	[79]
PUU	Covalent intrinsic	Disulfide exchange	Unfilled	[80,81]
Polyamide ionene	Combined intrinsic	Ionic interactions + Hydrogen bonds + *π*-*π* stacking	Unfilled	[82]
Ionic elastomer	Non-covalent intrinsic	Ionic interactions	Unfilled	[83]
SBR	Covalent intrinsic	Disulfide exchange	GTR ^1^	[78,84]
SBR	Covalent intrinsic	Disulfide exchange	dGTR ^2^	[85]
XNBR	Non-covalent intrinsic	Ionic interactions	GTR	[79]
Silicone elastomer	Covalent intrinsic	Thiol exchange	Ag nanoparticles	[86]

^1^ Ground tire rubber (GTR) from end-of-life tires. ^2^ Devulcanized ground tire rubber (dGTR) from end-of-life tires.

## Data Availability

Not applicable.

## References

[1] Wemyss A.M., Bowen C., Plesse C., Vancaeyzeele C., Nguyen G.T.M., Vidal F., Wan C. (2020). Dynamic crosslinked rubbers for a green future: A material perspective. Mater. Sci. Eng..

[2] Zhang Y., Zhang Z., Wemyss A.M., Wan C., Liu Y., Song P., Wang S. (2020). Effective thermal-oxidative reclamation of waste tire rubbers for producing high-performance rubber composites. ACS Sustain. Chem. Eng..

[3] Zedler Ł., Przybysz-Romatowska M., Haponiuk J., Wang S., Formela K. (2020). Modification of ground tire rubber—promising approach for development of green composites. J. Compos. Sci..

[4] Zedler Ł., Colom X., Cañavate J., Saeb M.R., Haponiuk J.T., Formela K. (2020). Investigating the impact of curing system on structure-property relationship of natural rubber modified with brewery by-product and ground tire rubber. Polymers.

[5] Hassan A.A., Zhang Z., Formela K., Wang S. (2021). Thermo-oxidative exfoliation of carbon black from ground tire rubber as potential reinforcement in green tires. Compos. Sci. Technol..

[6] Formela K., Kurańska M., Barczewski M. (2022). Recent advances in development of waste-based polymer materials: A review. Polymers.

[7] Ghorai S., Bhunia S., Roy M., De D. (2016). Mechanochemical devulcanization of natural rubber vulcanizate by dual function disulfide chemicals. Polym. Degrad. Stab..

[8] Aoudia K., Azem S., Ait Hocine N., Gratton M., Pettarin V., Seghar S. (2017). Recycling of waste tire rubber: Microwave devulcanization and incorporation in a thermoset resin. Waste Manag..

[9] de Sousa F.D.B., Scuracchio C.H., Hu G.-H., Hoppe S. (2017). Devulcanization of waste tire rubber by microwaves. Polym. Degrad. Stab..

[10] Seghar S., Asaro L., Rolland-Monnet M., Aït Hocine N. (2019). Thermo-mechanical devulcanization and recycling of rubber industry waste. Resour. Conserv. Recycl..

[11] Colom X., Cañavate J., Formela K., Shadman A., Saeb M.R. (2021). Assessment of the devulcanization process of EPDM waste from roofing systems by combined thermomechanical/microwave procedures. Polym. Degrad. Stab..

[12] Wemyss A.M., Ellingford C., Morishita Y., Bowen C., Wan C. (2021). Dynamic polymer networks: A new avenue towards sustainable and advanced soft machines. Angew. Chem. Int. Ed..

[13] Bosnjak N., Silberstein M.N. (2021). Pathways to tough yet soft materials. Science.

[14] Roy N., Bruchmann B., Lehn J.-M. (2015). DYNAMERS: Dynamic polymers as self-healing materials. Chem. Soc. Rev..

[15] Zhang B., De Alwis Watuthanthrige N., Wanasinghe S.V., Averick S., Konkolewicz D. (2022). Complementary dynamic chemistries for multifunctional polymeric materials. Adv. Fun. Mater..

[16] Xie Z., Hu B.-L., Li R.-W., Zhang Q. (2021). Hydrogen bonding in self-healing elastomers. ACS Omega.

[17] Huang Q., Liu Y., Li S., Zhu M., Hao T., Zhou Z., Nie Y. (2022). Blending polar rubber with polyurethane to construct self-healing rubber with multiple hydrogen bond networks. Polymer.

[18] Liu J., Xiao C., Tang J., Liu Y., Hua J. (2020). Construction of a dual ionic network in natural rubber with high self-healing efficiency through anionic mechanism. Ind. Eng. Chem. Res..

[19] Das M., Naskar K. (2021). Development, characterization, and applications of a unique self-healable elastomer: Exploring a facile metal-ligand interaction. Polymer.

[20] Huang J., Gong Z., Chen Y. (2022). A stretchable elastomer with recyclability and shape memory assisted self-healing capabilities based on dynamic disulfide bonds. Polymer.

[21] Chen X., Dam M.A., Ono K., Mal A., Shen H., Nutt S.R., Sheran K., Wudl F. (2002). A thermally re-mendable cross-linked polymeric material. Science.

[22] Chen X., Wudl F., Mal A.K., Shen H., Nutt S.R. (2003). New thermally remendable highly cross-linked polymeric materials. Macromolecules.

[23] Ying H., Zhang Y., Cheng J. (2014). Dynamic urea bond for the design of reversible and self-healing polymers. Nat. Commun..

[24] Zhang L., Wang H., Zhu Y., Xiong H., Wu Q., Gu S., Liu X., Huang G., Wu J. (2020). Electron-donating effect enabled simultaneous improvement on the mechanical and self-healing properties of bromobutyl rubber ionomers. ACS Appl. Mater. Interfaces.

[25] Peng T., Huang J., Gong Z., Ding J., Chen Y. (2021). Multiple cross-linked networks enhanced ENR-based composite with excellent self-healing properties. Polym. Adv. Technol..

[26] Yeh C.-M., Lin C.-H., Han T.-Y., Xiao Y.-T., Chen Y.-A., Chou H.-H. (2021). Disulfide bond and Diels–Alder reaction bond hybrid polymers with high stretchability, transparency, recyclability, and intrinsic dual healability for skin-like tactile sensing. J. Mater. Chem. A.

[27] Wang S., Urban M.W. (2021). Self-healable fluorinated copolymers governed by dipolar interactions. Adv. Sci..

[28] Gao X., Fan W., Zhu W., Jiuwei G., Zhang P., Wang C., Wang X., Xia H., Wang Z., Huang W. (2022). Tough and healable elastomers via dynamic integrated moiety comprising covalent and noncovalent interactions. Chem. Mater..

[29] Yilmaz D., Lansade D., Lewandowski S., Perraud S., Llevot A., Carlotti S. (2022). Combination of permanent hydrosilylation and reversible Diels–Alder reactions for self-healing poly(dimethylsiloxane) materials with enhanced ageing properties. Mater. Today Chem..

[30] Jing T., Heng X., Guifeng X., Li L., Li P., Guo X. (2022). Rapid self-healing and tough polyurethane based on the synergy of multi-level hydrogen and disulfide bonds for healing propellant microcracks. Mater. Chem. Front..

[31] Tee B.C.K., Wang C., Allen R., Bao Z. (2012). An electrically and mechanically self-healing composite with pressure- and flexion-sensitive properties for electronic skin applications. Nat. Nanotechnol..

[32] Le H.H., Böhme F., Sallat A., Wießner S., auf der Landwehr M., Reuter U., Stöckelhuber K.-W., Heinrich G., Radusch H.-J., Das A. (2017). Triggering the self-healing properties of modified bromobutyl rubber by intrinsically electrical heating. Macromol. Mater. Eng..

[33] Cerdan K., Van Assche G., van Puyvelde P., Brancart J. (2020). A novel approach for the closure of large damage in self-healing elastomers using magnetic particles. Polymer.

[34] Zhang Y., Khanbareh H., Roscow J., Pan M., Bowen C., Wan C. (2020). Self-healing of materials under high electrical stress. Matter.

[35] Guo H., Han Y., Zhao W., Yang J., Zhang L. (2020). Universally autonomous self-healing elastomer with high stretchability. Nat. Commun..

[36] White S.R., Sottos N.R., Geubelle P.H., Moore J.S., Kessler M.R., Sriram S.R., Brown E.N., Viswanathan S. (2001). Autonomic healing of polymer composites. Nature.

[37] Zhang W., Zheng Q., Ashour A., Han B. (2020). Self-healing cement concrete composites for resilient infrastructures: A review. Compos. Part B.

[38] Xu S., García A., Su J., Liu Q., Tabaković A., Schlangen E. (2018). Self-healing asphalt review: From idea to practice. Adv. Mater. Interfaces.

[39] van Dijk N., van der Zwaag S. (2018). Self-healing phenomena in metals. Adv. Mater. Interfaces.

[40] Wang S., Urban M.W. (2020). Self-healing polymers. Nat. Rev. Mater..

[41] Thakur V.K., Kessler M.R. (2015). Self-healing polymer nanocomposite materials: A review. Polymer.

[42] Kanu N.J., Gupta E., Vates U.K., Singh G.K. (2019). Self-healing composites: A state-of-the-art review. Compos. Part A.

[43] Huang J., Wróblewska A.A., Steinkoenig J., Maes S., Du Prez F.E. (2021). Assembling lipoic acid and nanoclay into nacre-mimetic nanocomposites. Macromolecules.

[44] Yang Y., Urban M.W. (2013). Self-healing polymeric materials. Chem. Soc. Rev..

[45] Blaiszik B.J., Kramer S.L.B., Olugebefola S.C., Moore J.S., Sottos N.R., White S.R. (2010). Self-healing polymers and composites. Annu. Rev. Mater. Res..

[46] Binder W.H. (2014). Self-Healing Polymers: From Principles to Applications.

[47] Bekas D.G., Tsirka K., Baltzis D., Paipetis A.S. (2016). Self-healing materials: A review of advances in materials, evaluation, characterization, and monitoring techniques. Compos. Part B.

[48] Hernández Santana M., den Brabander M., García S., van der Zwaag S. (2018). Routes to make natural rubber heal: A review. Polym. Rev..

[49] Utrera-Barrios S., Verdejo R., Lopez-Manchado M.A., Hernandez Santana M. (2020). Evolution of self-healing elastomers, from extrinsic to combined intrinsic mechanisms: A review. Mater. Horiz..

[50] Araujo-Morera J., López-Manchado M.A., Verdejo R., Hernández Santana M. (2022). Unravelling the effect of healing conditions and vulcanizing additives on the healing performance of rubber networks. Polymer.

[51] Behera P.K., Mohanty S., Gupta V.K. (2021). Self-healing elastomers based on conjugated diolefins: A review. Polym. Chem..

[52] Mashkoor F., Lee S.J., Yi H., Noh S.M., Jeong C. (2022). Self-healing materials for electronics applications. Int. J. Mol. Sci..

[53] Terryn S., Langenbach J., Roels E., Brancart J., Bakkali-Hassani C., Poutrel Q.-A., Georgopoulou A., George Thuruthel T., Safaei A., Ferrentino P. (2021). A review on self-healing polymers for soft robotics. Mater. Today.

[54] Cordier P., Tournilhac F., Soulie-Ziakovic C., Leibler L. (2008). Self-healing and thermoreversible rubber from supramolecular assembly. Nature.

[55] Sattar M.A., Patnaik A. (2020). Design principles of interfacial dynamic bonds in self-healing materials: What are the parameters?. Chem.-Asian J..

[56] Kohjiya S., Ikeda Y. (2014). Chemistry, Manufacture, and Applications of Natural Rubber.

[57] Men X., Wang F., Chen G.-Q., Zhang H.-B., Xian M. (2019). Biosynthesis of natural rubber: Current state and perspectives. Int. J. Mol. Sci..

[58] Toki S., Fujimaki T., Okuyama M. (2000). Strain-induced crystallization of natural rubber as detected real-time by wide-angle X-ray diffraction technique. Polymer.

[59] Amnuaypornsri S., Sakdapipanich J., Toki S., Hsiao B.S., Ichikawa N., Tanaka Y. (2008). Strain-induced crystallization of natural rubber: Effect of proteins and phospholipids. Rubber Chem. Technol..

[60] Carretero–González J., Ezquerra T.A., Amnuaypornsri S., Toki S., Verdejo R., Sanz A., Sakdapipanich J., Hsiao B.S., López–Manchado M.A. (2010). Molecular dynamics of natural rubber as revealed by dielectric spectroscopy: The role of natural cross–linking. Soft Matter.

[61] Amnuaypornsri S., Toki S., Hsiao B.S., Sakdapipanich J. (2012). The effects of endlinking network and entanglement to stress–strain relation and strain-induced crystallization of un-vulcanized and vulcanized natural rubber. Polymer.

[62] Toki S., Che J., Rong L., Hsiao B.S., Amnuaypornsri S., Nimpaiboon A., Sakdapipanich J. (2013). Entanglements and networks to strain-induced crystallization and stress–strain relations in natural rubber and synthetic polyisoprene at various temperatures. Macromolecules.

[63] Toki S., Kohjiya S., Ikeda Y. (2014). 5-The effect of strain-induced crystallization (SIC) on the physical properties of natural rubber (NR). Chemistry, Manufacture, and Applications of Natural Rubber.

[64] Tanasi P., Hernández Santana M., Carretero-González J., Verdejo R., López-Manchado M.A. (2019). Thermo-reversible crosslinked natural rubber: A Diels-Alder route for reuse and self-healing properties in elastomers. Polymer.

[65] Utrera-Barrios S., Hernández Santana M., Verdejo R., López-Manchado M.A. (2020). Design of rubber composites with autonomous self-healing capability. ACS Omega.

[66] Hernández M., Bernal M.M., Grande A.M., Zhong N., van der Zwaag S., García S.J. (2017). Effect of graphene content on the restoration of mechanical, electrical, and thermal functionalities of a self-healing natural rubber. Smart Mater. Struct..

[67] Hernández M., Grande A.M., Dierkes W., Bijleveld J., van der Zwaag S., García S.J. (2016). Turning vulcanized natural rubber into a self-healing polymer: Effect of the disulfide/polysulfide ratio. ACS Sustainable Chem. Eng..

[68] Hernández M., Grande A.M., van der Zwaag S., Garcia S.J. (2016). Monitoring network and interfacial healing processes by broadband dielectric spectroscopy: A case study on natural rubber. ACS Appl. Mater. Interfaces.

[69] Baker C.S.L., Gelling I.R., Newell R. (1985). Epoxidized natural rubber. Rubber Chem. Technol..

[70] Arroyo M., López-Manchado M.A., Valentín J.L., Carretero J. (2007). Morphology/behaviour relationship of nanocomposites based on natural rubber/epoxidized natural rubber blends. Compos. Sci. Technol..

[71] Sengloyluan K., Sahakaro K., Dierkes W.K., Noordermeer J.W.M. (2014). Silica-reinforced tire tread compounds compatibilized by using epoxidized natural rubber. Eur. Polym. J..

[72] Cao L., Yuan D., Xu C., Chen Y. (2017). Biobased, self-healable, high strength rubber with tunicate cellulose nanocrystals. Nanoscale.

[73] Huang J., Cao L., Yuan D., Chen Y. (2018). Design of novel self-healing thermoplastic vulcanizates utilizing thermal/magnetic/light-triggered shape memory effects. ACS Appl. Mater. Interfaces.

[74] Nie J., Mou W., Ding J., Chen Y. (2019). Bio-based epoxidized natural rubber/chitin nanocrystals composites: Self-healing and enhanced mechanical properties. Compos. Part B.

[75] Xu C., Nie J., Wu W., Zheng Z., Chen Y. (2019). Self-healable, recyclable, and strengthened epoxidized natural rubber/carboxymethyl chitosan biobased composites with hydrogen bonding supramolecular hybrid networks. ACS Sustain. Chem. Eng..

[76] Nie J., Fan J., Gong Z., Xu C., Chen Y. (2021). Frame-structured and self-healing ENR-based nanocomposites for strain sensors. Eur. Polym. J..

[77] Yikmis M., Steinbüchel A. (2012). Historical and recent achievements in the field of microbial degradation of natural and synthetic rubber. Appl. Environ. Microbiol..

[78] Hernández Santana M., Huete M., Lameda P., Araujo J., Verdejo R., López-Manchado M.A. (2018). Design of a new generation of sustainable SBR compounds with good trade-off between mechanical properties and self-healing ability. Eur. Polym. J..

[79] Utrera-Barrios S., Araujo-Morera J., Pulido de Los Reyes L., Verdugo Manzanares R., Verdejo R., López-Manchado M.Á., Hernández Santana M. (2020). An effective and sustainable approach for achieving self-healing in nitrile rubber. Eur. Polym. J..

[80] Martin R., Rekondo A., Ruiz de Luzuriaga A., Cabañero G., Grande H.J., Odriozola I. (2014). The processability of a poly(urea-urethane) elastomer reversibly crosslinked with aromatic disulfide bridges. J. Mater. Chem. A.

[81] Rekondo A., Martin R., Ruiz de Luzuriaga A., Cabañero G., Grande H.J., Odriozola I. (2014). Catalyst-free room-temperature self-healing elastomers based on aromatic disulfide metathesis. Mater. Horiz..

[82] O’Harra K., Sadaba N., Irigoyen M., Ruipérez F., Aguirresarobe R., Sardon H., Bara J. (2020). Nearly perfect 3D structures obtained by assembly of printed parts of polyamide ionene self-healing elastomer. ACS Appl. Polym. Mater..

[83] Aboudzadeh A., Fernandez M., Muñoz M.E., Santamaría A., Mecerreyes D. (2014). Ionic supramolecular networks fully based on chemicals coming from renewable sources. Macromol. Rapid Commun..

[84] Araujo-Morera J., Hernández Santana M., Verdejo R., López-Manchado M.A. (2019). Giving a second opportunity to tire waste: An alternative path for the development of sustainable self-healing styrene–butadiene rubber compounds overcoming the magic triangle of tires. Polymers.

[85] Alonso Pastor L.E., Núñez Carrero K.C., Araujo-Morera J., Hernández Santana M., Pastor J.M. (2022). Setting relationships between structure and devulcanization of ground tire rubber and their effect on self-healing elastomers. Polymers.

[86] Martín R., Rekondo A., Echeberria J., Cabañero G., Grande H.J., Odriozola I. (2012). Room temperature self-healing power of silicone elastomers having silver nanoparticles as crosslinkers. Chem. Commun..

[87] Utrera-Barrios S., Verdugo Manzanares R., Araujo-Morera J., González S., Verdejo R., López-Manchado M.Á., Hernández Santana M. (2021). Understanding the molecular dynamics of dual crosslinked networks by dielectric spectroscopy. Polymers.

[88] Aguirresarobe R.H., Nevejans S., Reck B., Irusta L., Sardon H., Asua J.M., Ballard N. (2021). Healable and self-healing polyurethanes using dynamic chemistry. Prog. Polym. Sci..

[89] Formoso E., Asua J.M., Matxain J.M., Ruipérez F. (2017). The role of non-covalent interactions in the self-healing mechanism of disulfide-based polymers. Phys. Chem. Chem. Phys..

[90] Irigoyen M., Fernández A., Ruiz A., Ruipérez F., Matxain J.M. (2019). Diselenide bonds as an alternative to outperform the efficiency of disulfides in self-healing materials. J. Org. Chem..

